# Clinical utility of comprehensive genomic profiling versus Oncomine Dx target test in pathological stage II–III non-small cell lung cancer

**DOI:** 10.1038/s41598-025-21559-5

**Published:** 2025-10-27

**Authors:** Kaito Yano, Kaoru Kaseda, Kohei Nakamura, Yu Okubo, Kyohei Masai, Tomoyuki Hishida, Shigenari Nukaga, Keiko Ohgino, Hideki Terai, Hiroyuki Yasuda, Yutaka Kurebayashi, Koichi Fukunaga, Hiroshi Nishihara, Keisuke Asakura

**Affiliations:** 1 Division of Thoracic Surgery, Department of Surgery, Keio University School of Medicine, 35 Shinanomachi, Shinjuku-ku, Tokyo, 160-8582 Japan; 2 Center for Cancer Genomics, Keio University School of Medicine, Tokyo, Japan; 3 Division of Pulmonary Medicine, Department of Internal Medicine, Keio University School of Medicine, Tokyo, Japan; 4 Department of Pathology, Keio University School of Medicine, Tokyo, Japan

**Keywords:** Non-small cell lung cancer, Comprehensive genomic profiling, Pathological stage, Driver gene mutations, Adjuvant therapy, Cancer, Oncology

## Abstract

The next-generation sequencing (NGS)–based Oncomine Dx Target Test (ODxTT) is the standard tool for guiding postoperative adjuvant therapy in patients with non-small cell lung cancer (NSCLC) in Japan. To advance precision oncology, we evaluated the clinical utility of an in-house comprehensive genomic profiling (CGP) assay, Rapid-Neo, as a complementary approach to ODxTT in surgically resected NSCLC. Patients with pathological stage II–III NSCLC who underwent anatomical surgical resection between December 2019 and May 2024 were included. Resected specimens underwent genomic analysis using both ODxTT and Rapid-Neo CGP. We evaluated the mutational concordance and the frequency of additional actionable alterations identified by CGP. Among 68 eligible patients, driver mutation results were concordant in 64 (94.1%) cases. Crucially, CGP rescued one patient for targeted therapy by detecting an EGFR mutation where ODxTT failed due to insufficient DNA. CGP also identified rare *EGFR* variants not covered by ODxTT in two cases, although it failed to detect a *RET* fusion in one patient. Furthermore, CGP revealed additional actionable alterations, such as tumor suppressor gene mutations, in 57 patients (83.8%). Our in-house CGP shows high concordance with ODxTT and serves as a powerful complementary tool. These findings support a strategic testing algorithm where CGP is incorporated for patients with negative or inconclusive ODxTT results, or at the time of recurrence, to maximize opportunities for individualized therapy in resected NSCLC.

## Introduction

Non-small cell lung cancer (NSCLC) is considered resectable in approximately 30% of cases^1,2^. However, patients with stage II or more advanced disease have a high risk of postoperative recurrence^3^. In individuals with pathological (p-) stage II–IIIA NSCLC, postoperative platinum-based chemotherapy has been shown to improve the 5-year survival rate by approximately 5%^4–6^, leading to its widespread adoption.

Recent evidence underscores the importance of tailoring adjuvant therapy based on PD-L1 expression and driver mutation status. The IMpower010 trial has demonstrated the efficacy of immune checkpoint inhibitors (ICIs) in patients who lack driver mutations and exhibit programmed death ligand 1 (PD-L1) expression of at least 1%^7^. Furthermore, studies have reported favorable outcomes with the postoperative ICI–chemotherapy combinations^8,9^. Additionally, the ADAURA trial in *EGFR*-positive NSCLC and the ALINA trial in *ALK*-positive NSCLC have highlighted the survival benefits of targeted adjuvant therapy in eligible patients^10,11^.

Given these findings, early postoperative assessment of PD-L1 expression and driver alterations (e.g., *EGFR*, *ALK*) is now recommended for patients with the p-stage II or higher NSCLC to optimize adjuvant therapy selection. In Japan, the Oncomine Dx Target Test (ODxTT) has been covered by national insurance as a next-generation sequencing (NGS)–based panel for comprehensive driver mutation testing and companion diagnostics^12^. For p-stage II and above, early postoperative genetic testing—including PD-L1 assessment—has become standard clinical practice.

Meanwhile, precision oncology using comprehensive genomic profiling (CGP) for multiple cancer-related genes has gained significant attention. Since June 2019, Japan has provided insurance coverage for genomic panel tests (OncoGuide™ NCC Oncopanel System, FoundationOne^®^ CDx (F1CDx)), which enable multi-gene analysis at a relatively low cost, marking a pivotal step toward integrating genomic medicine into routine oncology care. However, these insured CGP tests are restricted to patients who either lack standard treatment options or have already completed standard therapy, limiting their clinical impact. Consequently, only approximately 10% of patients ultimately receive genotype-matched therapy^13,14^.

To address these limitations, Keio University Hospital introduced a proprietary CGP test, Rapid-Neo, in January 2022 as part of routine clinical practice (approval no. 776200). Unlike existing insured CGP tests, Rapid-Neo CGP imposes no restrictions on patient eligibility or testing timelines, potentially increasing both the uptake of molecular profiling and the likelihood that genomic findings will directly inform subsequent treatment strategies. This approach is designed to expand patient access to precision medicine, ensuring that more individuals can benefit from biomarker-driven therapeutic interventions.

At our institution, Rapid-Neo CGP testing has been introduced for patients with p-stage II–III NSCLC following surgical resection. While ODxTT is a targeted panel designed to detect specific, well-established driver alterations in approximately 46 genes, our in-house Rapid-Neo CGP provides a broader genomic landscape by analyzing 143 cancer-related genes for a wider range of alterations, including single-nucleotide variants, copy number alterations, and tumor suppressor gene mutations. By detecting driver mutations not covered by ODxTT, this test may provide clinically valuable molecular insights for treatment selection in the event of disease recurrence. Additionally, identifying actionable genetic alterations associated with ICI resistance, such as *STK11* and *KEAP1*^15^, as well as mutations in tumor-suppressor genes (TSGs) like *TP53* and *RB1*^16^, could aid in predicting the efficacy of adjuvant chemotherapy and informing patient prognosis. However, when implementing precision medicine in early postoperative settings, ensuring alignment with existing companion diagnostic NGS tests remains essential for maintaining clinical and regulatory consistency.

This study aimed to compare the diagnostic and clinical utility of the Rapid-Neo CGP test with the existing ODxTT in surgically resected NSCLC cases and to clarify their mutational concordance and complementary roles. In cases of discordant findings, potential underlying causes were systematically investigated. Furthermore, by applying precision medicine approaches to postoperative patients—rather than restricting CGP to those with unresectable or treatment-refractory disease—this study sought to elucidate, in real-world settings, the frequency of clinically actionable or druggable genetic alterations and their correlation with patient-specific clinical factors.

## Patients and methods

### Participants

This study included patients with p-stage II–III NSCLC who underwent anatomical surgical resection at our institution between December 2019 and May 2024. Patient eligibility was based on the 9th edition of the TNM classification^17^. At our institution during this period, the ODxTT was performed for all surgically resected p-Stage II–III patients who provided informed consent for the test to guide postoperative adjuvant therapy decisions. Furthermore, beginning in January 2022, our in-house CGP test, Rapid-Neo, was additionally offered to patients who provided separate consent as part of an institutional advanced medical care program. For this study, we selected the cohort of patients whose resected specimens underwent genomic analysis using both ODxTT and Rapid-Neo CGP. Clinical data, including age, sex, smoking history, tumor characteristics, histopathological findings, and genomic analysis results, were extracted from electronic medical records.

### ODxTT

The ODxTT was performed by LSI Medience Corporation (Tokyo, Japan). As of the end of 2024, ODxTT had been approved in Japan as a companion diagnostic for mutations in *EGFR*, *BRAF*, and *HER2*, as well as *ALK*, *ROS1*, and *RET* fusions and *MET* exon 14 skipping alterations.

All specimens analyzed using ODxTT were formalin-fixed, paraffin-embedded (FFPE) tumor tissue. The ODxTT assay detects 46 target gene mutations from tumor-derived DNA and 21 target gene fusions from tumor-derived RNA. Tumor cell content (TCC) was defined as the percentage of tumor cells relative to all nucleated cells in the specimen, with a minimum TCC of 30% typically required for ODxTT analysis. In cases where TCC ranged between 10% and 30%, macrodissection was considered to enrich tumor cell content. NGS analysis was conducted on 5-µm FFPE tissue Sect. (10 slides in total). If the tissue surface area measured between 1 and 4 mm^2^, a minimum of 15 slides was prepared. The minimum required amount of extracted DNA and RNA was 10 ng each.

### Rapid-Neo

In January 2022, we introduced Rapid-Neo, an in-house CGP system, as part of routine clinical practice. All sequencing procedures were conducted on-site at our hospital, following a previously reported method with slight modifications^18,19^. The Rapid-Neo CGP panel analyzes tumor-derived DNA for single-nucleotide variants, small insertions and deletions (indels), copy number alterations (CNAs), and 11 types of gene fusions, covering 143 cancer-related genes (Supplementary Table S1).

For CNA analysis, loss of heterozygosity (LOH) was classified based on a deviation exceeding 2 standard deviations from the baseline. Unstained FFPE Sects. (10–20 slides, each 5 μm thick) were used, requiring a minimum of 20 ng of extracted DNA. Genetic analysis required a TCC of at least 20%. DNA integrity was assessed using the DNA integrity number (DIN), and samples with a DIN score > 2.0 were forwarded to library preparation for genomic sequencing. Target-capture sequencing was performed on a NextSeq 550 platform (Illumina, San Diego, CA, USA).

### Details of genomic analysis

In this study, the turnaround time (TAT) was defined as the period from specimen label submission to result reporting. With Rapid-Neo CGP, in addition to measuring the pathological TCC in FFPE samples, the genomic TCC was also assessed. The genomic TCC was calculated using a bioinformatics algorithm that evaluates variant allele frequency (VAF), CNAs, and LOH. Furthermore, the DNA read depth during CGP testing was measured. In this study, we defined “actionable mutations” as genomic alterations linked by approved therapies or strong clinical evidence to therapeutic response, prognosis, or resistance. This definition includes key tumor suppressor genes and biomarkers (e.g., *TP53*, *RB1*, *STK11*, *KEAP1*) that can guide clinical decisions in NSCLC, even without a directly associated targeted drug.

### Statistical analyses

The association between the two groups was evaluated using the Pearson correlation coefficient. Statistical analyses were performed using R statistical software, version 3.6.0 (R Foundation for Statistical Computing, Vienna, Austria).

## Results

### Patient characteristics

During the study period, 777 patients underwent surgical resection for lung cancer. Among them, 140 patients were diagnosed with p-stage II–III NSCLC. Of these, 71 patients underwent ODxTT. Among them, the 68 patients who additionally underwent Rapid-Neo CGP were included in this study. Patient characteristics are summarized in Table 1. The median age was 69 years, and 36 patients (52.9%) were men. A history of smoking was documented in 47 patients (69.1%). Adenocarcinoma was the predominant histological subtype, identified in 50 patients (73.5%).

**Table 1:** Patient background and characteristics (*n* = 68).

Characteristics	Value
Age (years)	69 [27–88]
Sex	
Male	36 (52.9)
Female	32 (47.1)
Smoking habit	
Present	47 (69.1)
Absent	21 (30.9)
Histology	
Adenocarcinoma	50 (73.5)
Adenoid cystic carcinoma	1 (1.5)
Squamous cell carcinoma	6 (8.8)
Adenosquamous carcinoma	2 (2.9)
LCNEC	4 (5.9)
Large cell carcinoma	1 (1.5)
Sarcomatoid	4 (5.9)
pN	
0	23 (32.4)
1	28 (41.2)
2a	13 (19.1)
2b	4 (5.9)
p-Stage	
IIA	22 (32.4)
IIB	22 (32.4)
IIIA	21 (30.9)
IIIB	3 (4.4)
PD-L1	
≥ 50%	15 (22.1)
1%–49%	32 (47.1)
< 1%	21 (30.9)
Postoperative adjuvant therapy	
Present	24 (35.3)
UFT	2 (2.9)
Platinum-based only	9 (13.2)
ICI combined	6 (8.8)
TKI combined	7 (10.3)
Absent	44 (64.7)

Values are presented as median [range] or n (%).

ICI, immune checkpoint inhibitor; LCNEC, large cell neuroendocrine carcinoma; pN, pathological N; p-Stage, pathological stage; PD-L1, programmed death ligand 1; TKI, tyrosine kinase inhibitor; UFT, tegafur-uracil.

## Genomic analysis

The genomic testing details for ODxTT and Rapid-Neo CGP are presented in Table 2. The median TAT was 10 days for ODxTT and 28 days for Rapid-Neo CGP. For Rapid-Neo CGP, the median pathological TCC was 50%, whereas the median genomic TCC was 40%, showing a positive correlation (*r* = 0.489). The median DNA sequencing depth for Rapid-Neo CGP was 236.

**Table 2:** Details on genomic testing with ODxTT and Rapid-Neo CGP (*n* = 68).

Factors	Value
TAT (days)	
ODxTT	10 [5–19]
CGP	28 [15–45]
TCC on CGP analysis	
Pathology	50 [30–80]
Genomic	40 [10–80]
Correlation coefficient	0.489
DNA depth on CGP analysis	236 [76–416]

Values are presented as median [range], except for the correlation coefficient.

CGP, comprehensive genomic profiling; ODxTT, Oncomine Dx Target Test; TAT, turnaround time; TCC, tumor cell content.

### Rapid-Neo CGP results

**Fig. 1:**
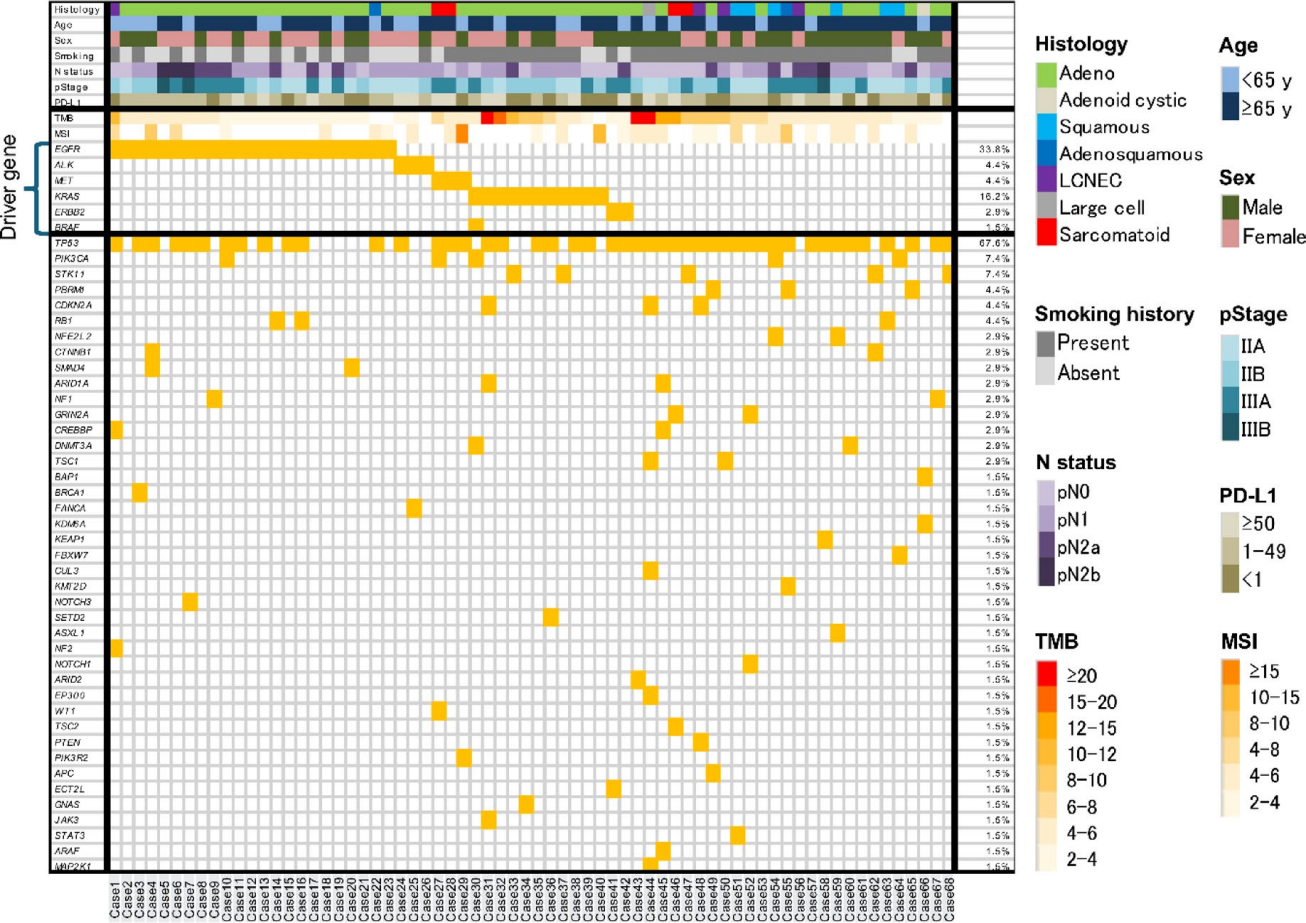
Heatmap illustrating the results of Rapid-Neo CGP analysis for all 68 patients. Sequencing was successfully performed in all cases. TMB and MSI status are also presented. TMB is shown as a continuous variable, with darker colors indicating higher values. A cutoff of 10 mutations per megabase (mut/Mb) was used to define TMB-high. MSI is also shown as a continuous variable, with darker colors indicating higher percentages of instability. MSI-high is defined as ≥ 12.5% instability, and MSS as < 12.5%. Driver mutations were identified in 42 patients (61.8%). Notably, even among patients without detectable driver mutations, a substantial number exhibited diverse genetic alterations. CGP, comprehensive genomic profiling; MSI, microsatellite instability; MSS, microsatellite stable; TMB, tumor mutational burden.

Figure 1 presents the heatmap of the Rapid-Neo CGP results for all 68 patients. Sequencing was successful in all cases, achieving a 100% success rate. Forty-two patients (61.8%) harbored at least one driver mutation. Even among driver gene–negative cases, many patients exhibited various genetic alterations.

### Concordance with ODxTT and CGP of druggable driver detection

**Fig. 2:**
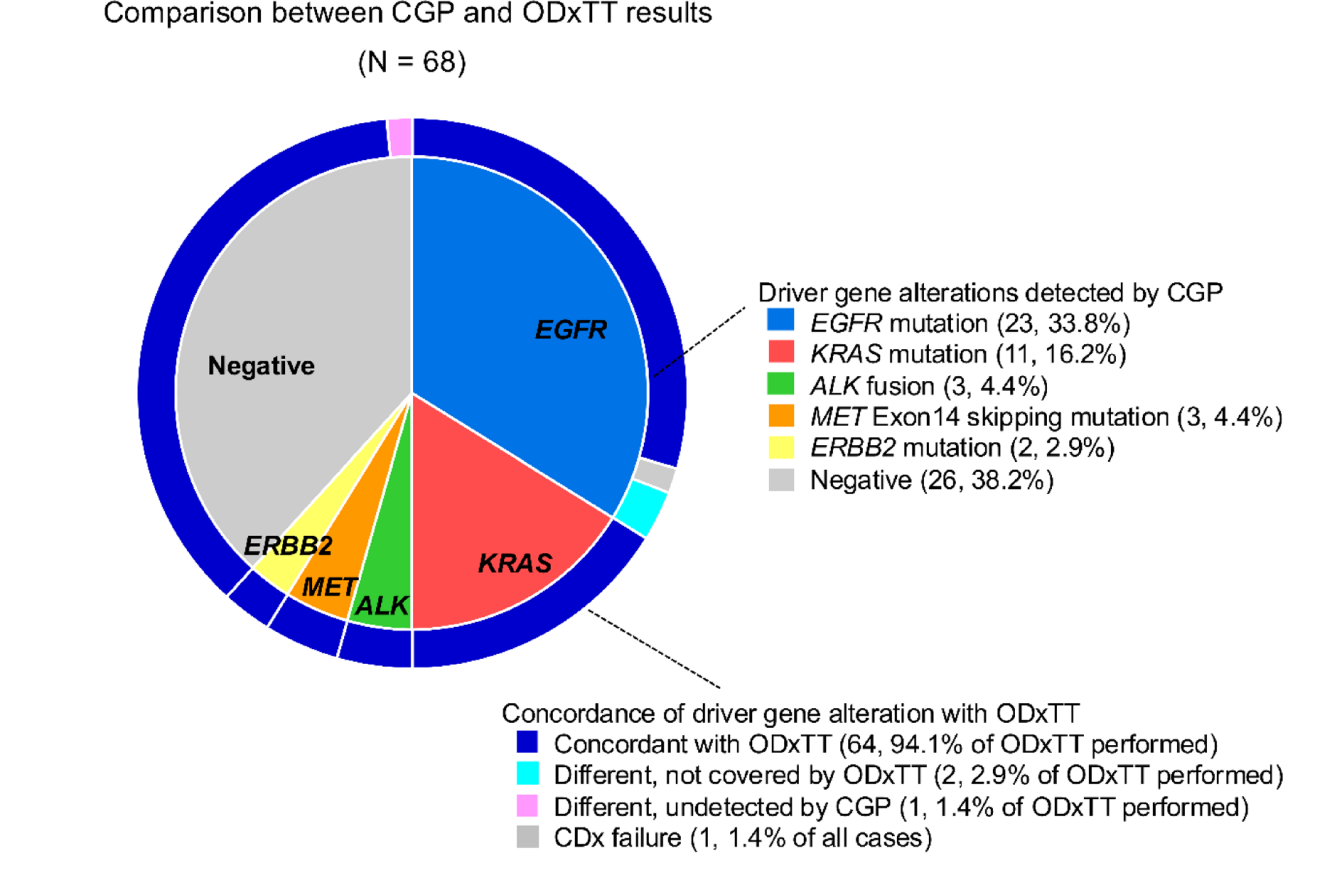
Pie chart comparing the detection of driver mutations between Rapid-Neo CGP and ODxTT. The inner section of the chart represents ODxTT results, whereas the outer section corresponds to Rapid-Neo CGP findings. Concordance was observed in 64 (94.1%) of 68 cases. Among the 4 discordant cases, 1 case failed ODxTT due to insufficient DNA, whereas CGP successfully confirmed *EGFR* exon 19 deletion positivity. In 2 cases classified as negative by ODxTT, Rapid-Neo CGP identified *EGFR* p.D770_H773dup and *EGFR* p.E114K mutations, respectively. Conversely, 1 case that tested positive for a *RET* fusion by ODxTT was negative on CGP analysis. CGP, comprehensive genomic profiling; ODxTT, Oncomine Dx Target Test.

With Rapid-Neo CGP, *EGFR* alterations were found in 23 patients (33.8%), *ALK* fusions in 3 patients (4.4%), *KRAS* mutations in 11 patients (16.2%), and *MET* exon 14 skipping mutations in 3 patients (4.4%). *ERBB2* mutations were identified in 2 patients (2.9%). A total of 26 patients (38.2%) tested negative for driver mutations on the CGP panel (summarized in Supplementary Table S2). Figure 2 compares the results of driver gene detection between Rapid-Neo CGP and ODxTT. Concordance was achieved in 64 (94.1%) of 68 cases. Of the 4 discordant cases, 1 failed the ODxTT due to insufficient DNA, although CGP confirmed *EGFR* exon 19 deletion positivity. In 2 cases considered negative on ODxTT, Rapid-Neo CGP identified *EGFR* p.D770_H773dup and *EGFR* p.E114K mutations, respectively. Conversely, a single case positive for a *RET* fusion by ODxTT tested negative on CGP.

### Actionable gene alterations identified by CGP

Table 3 highlights additional actionable genetic alterations identified by CGP. The most prevalent alteration was *TP53* mutations, detected in 46 patients (67.6%). *RB1* mutations were identified in 3 patients (4.4%), whereas *STK11* and *KEAP1* mutations were found in 7 (10.3%) and 2 (2.9%) patients, respectively. Additionally, 9 patients (13.2%) had a high tumor mutational burden (TMB-H), whereas 2 patients (2.9%) exhibited microsatellite instability-high (MSI-H) status.

**Table 3:** Actionable gene alterations and related information by CGP testing (*n* = 68).

Factors	Value
*TP53*	46 (67.6)
*RB1*	3 (4.4)
*STK11*	7 (10.3)
*KEAP1*	2 (2.9)
TMB-H	9 (13.2)
MSI-H	2 (2.9)

Values are presented as n (%).

CGP, comprehensive genomic profiling; MSI-H, microsatellite instability-high; TMB-H, tumor mutational burden-high.

### Tumor suppressor gene mutations on treatment outcomes in EGFR-mutant lung cancer

**Fig. 3:**
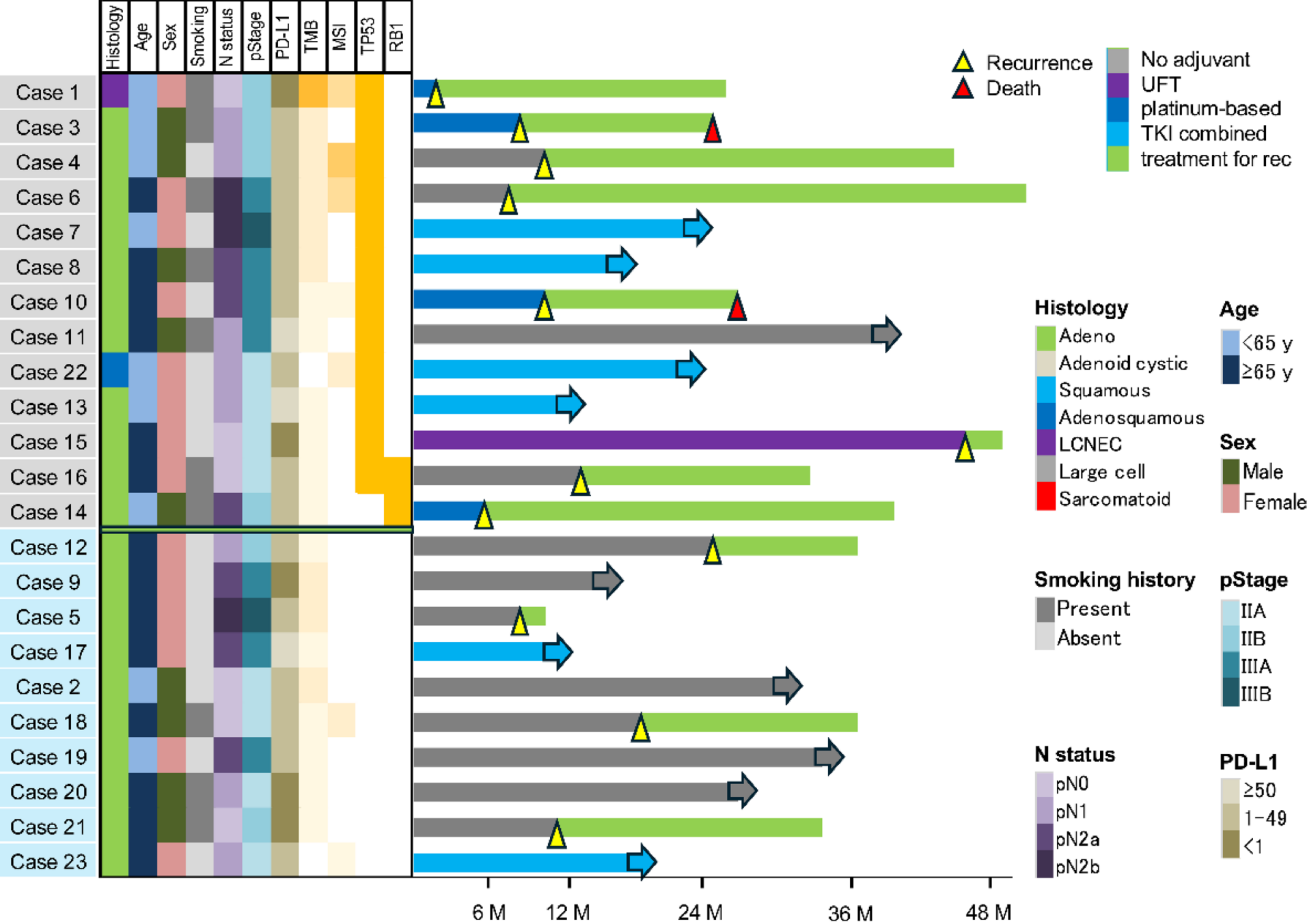
Swimmer plot illustrating treatment outcomes stratified by the presence or absence of TSG mutations in *EGFR*-positive cases. Cases under follow-up without recurrence are indicated by arrows. Among the 23 patients with *EGFR*-mutant NSCLC, *TP53* mutations were detected in 12 cases, whereas *RB1* mutations were identified in 2 cases (1 patient harbored both mutations). Among the 13 patients with TSG mutations, recurrence occurred in 8 cases, and 2 patients succumbed to disease progression. In contrast, among the 10 patients without TSG mutations, recurrence was observed in 4 cases, but no deaths were reported. Notably, 3 patients without TSG mutations remained RFS even in the absence of postoperative adjuvant therapy. Among the 6 patients who received postoperative osimertinib in combination with chemotherapy, all maintained RFS. TSG, tumor-suppressor genes; RFS, recurrence-free survival.

Figure 3 illustrates treatment outcomes based on the presence or absence of TSG mutations in *EGFR*-positive NSCLC cases. Among the 23 *EGFR*-mutant lung cancer patients, *TP53* mutations were detected in 12 cases, whereas *RB1* mutations were identified in 2 cases (with 1 patient harboring both mutations).

Among the 13 patients with TSG mutations, recurrence occurred in 8 cases (61.5%), and 2 patients had disease progression. In contrast, among 12 patients without TSG mutations, recurrence was observed in only 4 cases (33.3%), no deaths occurred, and 3 patients achieved recurrence-free survival (RFS) without postoperative adjuvant therapy.

Among the 6 patients who received postoperative osimertinib combined with chemotherapy, all remained recurrence-free, but the follow-up period was relatively short (12.2–23.9 months).

### Patient backgrounds, actionable mutations, and treatment outcomes in postoperative tyrosine kinase inhibitors, and ICI cases

Supplementary Table S3 summarizes the clinical backgrounds, actionable gene mutations, and treatment outcomes of 7 patients who received postoperative tyrosine kinase inhibitors (TKIs), including 6 cases treated with osimertinib and 1 case treated with alectinib. All patients maintained RFS throughout the observation period.

Supplementary Table S4 presents the clinical characteristics, genomic profiles, and treatment courses of 6 patients treated with ICIs postoperatively. *TP53* mutations were present in all ICI-treated cases, and 2 patients experienced recurrence despite adjuvant ICI therapy.

### A case with ODxTT analysis failure

**Fig. 4:**
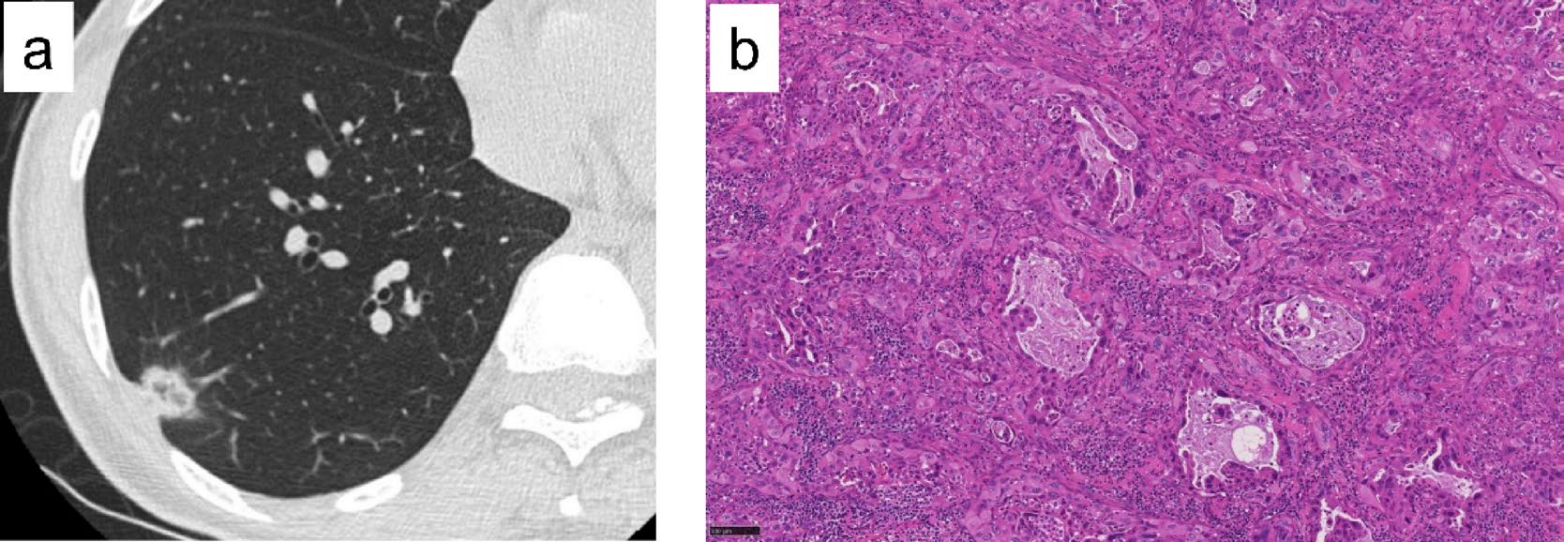
A 52-year-old woman. (**a**) Right lower lobectomy for a lung tumor located in the right lower lobe was performed. (**b**) The final pathological examination confirmed adenocarcinoma, classified as pathological T1bN1M0, stage IIB. In the resected tumor specimen, the TCC was 30%, and no driver mutations were identified by ODxTT. However, CGP detected an *EGFR* exon 19 deletion and postoperative adjuvant therapy with osimertinib was initiated. CGP, comprehensive genomic profiling; ODxTT, Oncomine Dx Target Test; TCC, tumor cell content.

A case demonstrating the clinical utility of CGP involved a 52-year-old woman who underwent a right lower lobectomy for a lung tumor (Fig. 4a). The final pathological examination confirmed adenocarcinoma, classified as pathological T1bN1M0, stage IIB. In the resected tumor specimen, the TCC was 30% (Fig. 4b), and the standard ODxTT did not identify any driver mutations. However, subsequent CGP successfully detected an *EGFR* exon 19 deletion, which was validated using the Cobas *EGFR* version 2.0 assay. Identifying this driver mutation by CGP directly altered the treatment plan, enabling the initiation of postoperative adjuvant therapy with osimertinib.

### Visual abstract legend

Rapid-Neo CGP showed high concordance with ODxTT and identified additional actionable mutations in pathological stage II–III NSCLC. It supports individualized treatment strategy after surgery.CGP, comprehensive genomic profiling; NSCLC, non-small cell lung cancer; ODxTT, Oncomine Dx Target Test

## Discussion

This study demonstrates that in-house Rapid-Neo CGP provides significant, complementary clinical value to the standard ODxTT for resected p-stage II-III NSCLC. While demonstrating high concordance (94.1%) in detecting major driver mutations, CGP enhanced clinical decision-making by identifying actionable mutations in cases where ODxTT failed or in variants not covered by the targeted panel. Furthermore, it provided a broader genomic landscape, revealing prognostic biomarkers and potential mechanisms of therapeutic resistance in the majority of patients.

Perhaps the most significant finding of this study is the demonstration of how CGP can complement existing companion diagnostics to rescue patients who might otherwise miss therapeutic opportunities. This was clearly exemplified by a notable case involving a patient initially classified as driver mutation–negative by ODxTT, yet in whom CGP subsequently detected an *EGFR* exon 19 deletion, enabling the administration of osimertinib as postoperative adjuvant therapy. The reason for this critical discrepancy often lies in the test’s sensitivity to low TCC. The *EGFR* false-negative rate for ODxTT is reported at approximately 6.1%^20^. In this instance, the patient’s TCC was 30%, precisely meeting the minimum threshold required for ODxTT analysis. In contrast, Rapid-Neo CGP requires only 20% TCC, thereby facilitating successful *EGFR* mutation detection and ensuring that the patient received targeted therapy based on their molecular profiling, despite the initial test’s limitations. Furthermore, Rapid-Neo CGP does not necessitate substantially greater DNA or tissue quantities compared with ODxTT. Although tissue volume is seldom a limiting factor in surgical specimens, performing CGP with minimal tissue requirements remains a distinct advantage of this in-house assay.

CGP identified *EGFR* p.D770_H773dup and *EGFR* p.E114K in 2 cases initially classified as driver gene–negative by ODxTT. These mutations are absent from the ODxTT panel of reportable variants. The *EGFR* p.D770_H773dup mutation is categorized as an exon 20 insertion, which has been associated with reduced responsiveness to both *EGFR* TKIs and ICIs^21^. Therefore, the ability to detect such mutations provides clinically valuable information that can directly influence future treatment strategies, especially upon disease recurrence. Though targeted therapies for *EGFR* exon 20 insertion mutations are approved for advanced disease, early detection remains crucial. Re-biopsy at recurrence is often infeasible, making the primary tumor’s genomic data an invaluable guide for immediate treatment decisions, ensuring patients can rapidly access effective therapies. The *EGFR* p.E114K variant is exceedingly rare, with unclear clinical significance. Previous studies have reported that among NSCLC patients initially classified as driver gene–negative, CGP subsequently identified novel driver gene alterations in approximately 24.5% of cases^22^. Further research and the accumulation of real-world clinical evidence are warranted to elucidate the impact of rare *EGFR* mutations on postoperative treatment strategies.

CGP also detected co-occurring *KRAS* p.A146T and *BRAF* p.N581L mutations in one case, whereas ODxTT failed to identify the latter. *BRAF* p.N581L is a class 3 non-V600E variant, and in this case, the *KRAS* alteration is likely the primary driver mutation. The combination of dabrafenib and trametinib has been reported to be effective against *BRAF* non-V600E mutations^23^. Thus, the detection of relatively rare driver mutations or co-occurring mutations through CGP may provide critical insights for exploring novel targeted therapies or combination regimens in the event of recurrence.

Conversely, in cases where ODxTT identified a *RET* fusion gene, CGP testing did not detect the alteration. In one such case, ODxTT identified a *RET* fusion gene with *KIF5B* as the fusion partner, a finding consistent with a previous report^24^. However, CGP detected only a single read, which did not meet the threshold for positivity and was consequently reported as negative. Given that DNA-based CGP has intrinsic limitations in detecting fusion genes or splicing abnormalities, with a fusion detection rate of approximately 73%^25^, it is essential to consider complementary molecular testing strategies. These may include integrating CGP with RNA-based assays such as ODxTT, revising the read-threshold criteria for DNA-based assays, or developing CGP methodologies incorporating RNA sequencing.

Our CGP analysis detected actionable genetic alterations in most cases, including those without identifiable driver mutations. Regarding TSG mutations, NSCLC harboring *TP53* mutations is associated with poor response to ICIs and worse overall prognosis^26,27^. Furthermore, *EGFR*-mutated tumors harboring *TP53* alterations exhibit reduced sensitivity to TKIs and are linked to unfavorable outcomes, with *TP53* mutations recognized as an independent adverse prognostic factor^28^. Additionally, *EGFR*-positive NSCLC with concurrent *TP53* and *RB1* mutations has been shown to be predisposed to histological transformation to small-cell lung cancer^16^. In our cohort, of the 10 cases without any TSG aberrations, 3 achieved long-term survival without postoperative adjuvant chemotherapy. In contrast, among cases with TSG aberrations, those who did not receive postoperative osimertinib experienced a relatively high recurrence rate. However, extended further follow-up is necessary to clarify the prognostic implications of these genetic alterations.

TMB-high status has been correlated with improved ICI responsiveness and favorable prognosis^29^. Even in patients harboring *TP53* mutations, TMB-high status may confer improved clinical outcomes^30^. In this study, 9 cases were identified as TMB-high. Among them, 1 patient with a *KRAS* G12C mutation, who also harbored a *TP53* mutation and high TMB, received postoperative adjuvant ICI therapy but experienced disease recurrence 15 months post-surgery. Conversely, *KEAP1* and *STK11* mutations have been implicated in resistance to ICIs^31^. The concurrent presence of *KRAS* mutations, frequently referred to as the “triple-negative phenotype,” has also been associated with reduced ICI efficacy^32^. Currently, adjuvant chemotherapy regimens incorporating ICIs are generally recommended irrespective of these genetic alterations. In this study, none of the patients who received postoperative adjuvant ICI therapy harbored these resistance-associated genetic mutations. Further investigations are warranted to elucidate the efficacy of ICI in the adjuvant setting and its interaction with specific genomic alterations and long-term prognosis.

Several practical limitations of implementing Rapid-Neo CGP in a routine clinical setting warrant discussion. Notably, our in-house CGP has a longer TAT compared to ODxTT (median: 28 vs. 10 days, Table 2). This extended TAT can be a challenge in the postoperative setting, where the timely initiation of adjuvant therapy is often critical. However, as our findings demonstrate, the clinical benefits provided by CGP may well outweigh these drawbacks in select patient populations. This value is multifaceted: first, CGP enables the identification of actionable targets and offers a potential lifeline for patients who are not eligible for targeted therapies based on standard tests; second, it offers crucial prognostic insights through biomarkers such as *TP53* mutations and TMB status; and third, it delivers a comprehensive genomic landscape that is essential for formulating treatment strategies in the event of disease recurrence. Therefore, for specific patient populations—such as those with ODxTT-negative results, younger patients, or those at high risk for recurrence—the investment in CGP is likely to yield clinical benefits that justify its longer TAT and additional cost.

It is important to compare our in-house Rapid-Neo assay with commercial CGP panels like F1CDx to contextualize its clinical value. Under the Japanese reimbursement system, the use of F1CDx is largely restricted to patients with advanced cancer who have exhausted standard therapies^14^. In contrast, our in-house test is free from these constraints, offering a unique advantage in delivering precision medicine to an underserved population: early-stage, post-surgical patients. Furthermore, as an on-site assay that eliminates the need for specimen transport, it also holds potential advantages in turnaround time and cost-effectiveness.

Several limitations of this study must be acknowledged. First, this investigation was conducted at a single institution using an in-house CGP, potentially limiting the generalizability of the findings. Second, it exclusively included Japanese patients; therefore, the study population was limited to individuals of East Asian descent. As a result, the generalizability of the findings to other racial and ethnic groups may be restricted. Third, the detection thresholds (e.g., minimum read counts) used in Rapid-Neo CGP, particularly for gene fusions, require further refinement and validation. Although this study demonstrated high concordance and clinical feasibility, larger prospective studies are essential to establish the prognostic and predictive significance of these additional genetic alterations in the perioperative setting. Finally, long-term survival and recurrence patterns have not yet been analyzed, and further follow-up is required to determine the clinical benefits of CGP-driven treatment modifications.

In conclusion, our findings suggest that CGP should not be viewed as a replacement for ODxTT, but rather as a complementary component within a strategic testing algorithm. ODxTT remains a valuable and rapid first-line test for initial postoperative decision-making to guide adjuvant therapy. Conversely, CGP e serves as a powerful second-line tool in select high-yield situations: for patients whose tumors are negative or inconclusive by ODxTT, and critically, at the time of disease recurrence to elucidate mechanisms of resistance and explore further therapeutic options. By integrating both testing modalities based on their respective strengths, clinicians can enhance individualized decision-making, ensuring that more patients with p-stage II–III NSCLC benefit from precision oncology across the continuum of care.

## Supplementary Information

Below is the link to the electronic supplementary material.

Supplementary Material 1

## Data Availability

The sequencing datasets generated during the current study have been deposited in the DDBJ Sequence Read Archive (DRA). The data are associated with BioProject accession PRJDB37491, BioSample accessions SAMD01671791–SAMD01671858, and Run accessions DRR728140–DRR728207. These datasets are publicly available at the following URL: https://ddbj.nig.ac.jp/resource/bioproject/PRJDB37491.
