# Supplementary material for: Clinical utility of comprehensive genomic profiling versus Oncomine Dx target test in pathological stage II–III non-small cell lung cancer
Source: Sci Rep. 2025 Oct 27;15:37477. doi: 10.1038/s41598-025-21559-5 (PMC12559205; doi:10.1038/s41598-025-21559-5)
Supplement: Supplementary file 1 — Supplementary Material 1 [file 41598_2025_21559_MOESM1_ESM.docx]

**Supplementary Table S1** The 143 genes detected by Rapid-Neo CGP

| *ALK* ^*^ | *BRAF* ^*^ | *EGFR* ^*^ | *ERBB2* ^*^ | *MET* ^*^ | *RET* ^*^ | *ROS1* ^*^ |  |
| --- | --- | --- | --- | --- | --- | --- | --- |
| *ABL1*^†^ | *AKT1*^†^ | *AR*^†^ | *AXL*^†^ | *CDK4*^†^ | *CTNNB1*^†^ | *DDR2*^†^ | *ERBB3*^†^ |
| *ERBB4*^†^ | *ESR1*^†^ | *FGFR1*^†^ | *FGFR2*^†^ | *FGFR3*^†^ | *GNA11*^†^ | *GNAQ*^†^ | *HRAS*^†^ |
| *IDH1*^†^ | *IDH2*^†^ | *JAK1*^†^ | *JAK2*^†^ | *JAK3*^†^ | *KIT*^†^ | *KRAS*^†^ | *MAP2K1*^†^ |
| *MAP2K2*^†^ | *MTOR*^†^ | *NRAS*^†^ | *NTRK1*^†^ | *NTRK2*^†^ | *NTRK3*^†^ | *PDGFRA*^†^ | *PIK3CA*^†^ |
| *RAF1*^†^ | *SMO*^†^ |  |  |  |  |  |  |
| *ACTN4* | *AKT2* | *AKT3* | *APC* | *ARAF* | *ARID1A* | *ARID2* | *ASXL1* |
| *ATM* | *ATRX* | *AXIN1* | *BAP1* | *BARD1* | *BCL2L11* | *BRCA1* | *BRCA2* |
| *BRIP1* | *CARD11* | *CCND1* | *CD274* | *CD79B* | *CDH1* | *CDK12* | *CDKN2A* |
| *CHEK2* | *CREBBP* | *CRKL* | *CSF1R* | *CUL3* | *DNMT3A* | *ECT2L* | *ENO1* |
| *EP300* | *EPCAM* | *EZH2* | *FANCA* | *FANCD2* | *FANCE* | *FBXW7* | *FGFR4* |
| *FH* | *FLT3* | *GNAS* | *GRIN2A* | *IGF1R* | *IGF2* | *IL7R* | *KDM6A* |
| *KDR* | *KEAP1* | *KMT2D* | *MAP2K4* | *MAP3K1* | *MAP3K4* | *MDM2* | *MDM4* |
| *MED12* | *MLH1* | *MSH2* | *MSH6* | *MYC* | *MYCN* | *MYD88* | *NF1* |
| *NF2* | *NFE2L2* | *NOTCH1* | *NOTCH2* | *NOTCH3* | *NPM1* | *NRG1* | *NT5C2* |
| *PALB2* | *PBRM1* | *PDGFRB* | *PIK3R1* | *PIK3R2* | *PMS2* | *POLD1* | *POLE* |
| *PRKCI* | *PTCH1* | *PTEN* | *RAC1* | *RAC2* | *RAD51C* | *RB1* | *RHOA* |
| *SETBP1* | *SETD2* | *SMAD4* | *SMARCA4* | *SMARCB1* | *SPOP* | *SRC* | *STAT3* |
| *STK11* | *TERT* | *TP53* | *TSC1* | *TSC2* | *VHL* | *WT1* | *XPC* |

^*^ Driver genes approved for companion diagnostics with ODxTT. ^†^ Genes also reported in ODxTT.

CGP, comprehensive genomic profiling; ODxTT, Oncomine Dx Target Test.

**Supplementary Table S2** Details of druggable driver genes identified by CGP testing (n=68)

| Factors | Value |
| --- | --- |
| *EGFR* (%) | 23 (33.8) |
| L858R | 12 (17.6) |
| Exon 19 deletion | 8 (11.8) |
| Uncommon | 3 (4.4) |
| *ALK* fusion (%) | 3 (4.4) |
| *KRAS* (%) | 11 (16.2) |
| G12C | 4 (5.9) |
| *MET* exon 14 skipping (%) | 3 (4.4) |
| *ERBB2* (%) | 2 (2.9) |
| Negative (%) | 26 (38.2) |

CGP, comprehensive genomic profiling.

**Supplementary Table S3** Cases in which TKI was combined with adjuvant chemotherapy

| Case no. | Age (years) | Sex | Histology | p-Stage | PD-L1 | ODxTT | CGP | Clinical course |
| --- | --- | --- | --- | --- | --- | --- | --- | --- |
| 7 | 48 | Female | Ad | IIIB | 1 | *EGFR* ex19del | *EGFR* p.E746_A750del  *TP53* p.G245S  *NOTCH3* p.L1912Wfs*7 | Osimertinib  24M DFS |
| 8 | 65 | Male | Ad | IIIA | <1 | *EGFR* L858R | *EGFR* L858R  *TP53* p.K292Nfs | Osimertinib  17M DFS |
| 13 | 52 | Female | Ad | IIA | 85 | *EGFR* ex19del | *EGFR* p.L747_A750delinsP  *TP53* p.Q192_H193delinsHY | Osimertinib  13M DFS |
| 17 | 73 | Female | Ad | IIIA | 30 | *EGFR* L858R | *EGFR* p.L858R | Osimertinib  12M DFS |
| 22 | 52 | Female | Ad-sq | IIA | 10 | Analysis failure | *EGFR* p.E746_A750del  *TP53* p.R273L | Osimertinib  23M DFS |
| 23 | 67 | Female | Ad | IIA | 1 | *EGFR* L858R | *EGFR* p.L858R | Osimertinib  19M DFS |
| 25 | 50 | Female | Ad | IIA | 50 | *ALK* fusion | *ALK* fusion  *FANCA* p.T329Nfs*10 | Alectinib  10M DFS |

Ad, adenocarcinoma; Ad-sq, adenosquamous carcinoma; CGP, comprehensive genomic profiling; DFS, disease-free survival; ex19del, exon 19 deletion; M, month; ODxTT, Oncomine Dx Target Test; PD-L1, programmed death ligand 1; p-Stage, pathological stage; TKI, tyrosine kinase inhibitor.

**Supplementary Table S4** Cases in which ICI was combined with adjuvant chemotherapy

| Case | Age (years) | Sex | Histology | p-Stage | PD-L1 | ODxTT | CGP | Clinical course |
| --- | --- | --- | --- | --- | --- | --- | --- | --- |
| 24 | 59 | Female | Ad | IIA | 80 | *ALK* fusion | *ALK* fusion  *TP53* p.Y236C | Atezolizumab  17M DFS |
| 32 | 56 | Female | Ad | IIA | 90 | *KRAS* G12C | *KRAS* p.G12C  *TP53* p.Q38Kfs*6  TMB high | Atezolizumab  Recurrence at 15M |
| 42 | 41 | Male | Ad | IIIA | 60 | *ERBB2* | *ERBB2*  *TP53* p.E287Rfs*58 | Atezolizumab  Recurrence at 16M |
| 61 | 70 | Male | Ad | IIIA | 100 | negative | *TP53* p.V272M | Atezolizumab  12M DFS |
| 65 | 69 | Male | Ad | IIB | 5 | negative | *TP53* p.R158L  *PRRM* p.R1027* | Atezolizumab  17M DFS |
| 67 | 74 | Male | Ad | IIB | 55 | negative | *TP53* p.R156P  *NF1* p.E1516 | Atezolizumab  20M DFS |

Ad, adenocarcinoma; CGP, comprehensive genomic profiling; DFS, disease-free survival; ICI, immune checkpoint inhibitor; M, month; ODxTT, Oncomine Dx Target Test; PD-L1, programmed death ligand 1; p-Stage, pathological stage.
